# Topologies of Nanoscale Droplets upon Head-On Collision from Large Molecular Dynamics Simulations

**DOI:** 10.1021/acs.langmuir.4c04588

**Published:** 2025-01-08

**Authors:** Leonie Tugend, Simon Homes, Jadran Vrabec

**Affiliations:** Thermodynamik, Technische Universität Berlin, 10587 Berlin, Germany

## Abstract

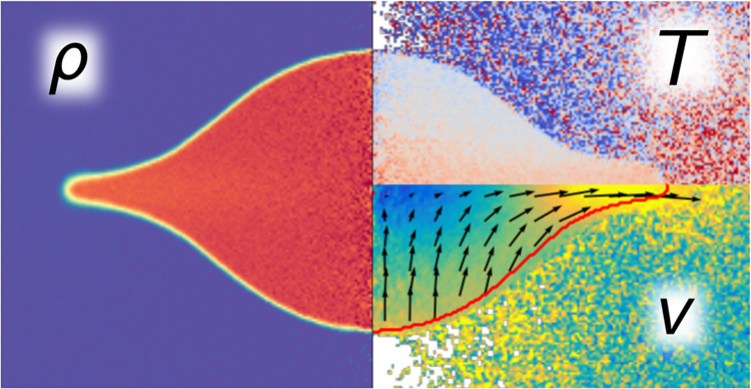

The binary collision of nanoscale droplets is studied with molecular dynamics simulation for droplets consisting of up to 2 × 10^7^ molecules interacting via a truncated and shifted form of the Lennard-Jones potential. Considering head-on collisions of droplets with a temperature near the triple point that occur in a saturated vapor of the same fluid, this work explores a range of collision topologies. Four droplet sizes, with a radius ranging from 30 to 120 molecule diameters, are simulated with a varying initial relative collision velocity, covering 36 cases in total. Due to the relatively large size of the droplets, this study aims to resolve the differences in the collision behavior between droplets on the micro- and on the macroscale. By analyzing various metrics of the impact, four distinct collision regimes are found: coalescence, stable collision, holes and shattering. Coalescence, observed at low Weber and Reynolds numbers, is the formation of a stable droplet without significant deformations of the merging objects. Stable collisions, characterized by the formation of one stable droplet with notable deformations during collision, occur within a Weber number range between 10 and 505. The holes regime is only observed for droplet radii greater than 30 molecule diameters and a Weber number between 505 to 750, while collision cases surpassing this Weber number fall into the shattering regime, resulting in the breakup into satellite structures.

## Introduction

Droplet collision processes play an important role in natural sciences^1^ and engineering.^2−4^ The collision of liquid droplets influences processes like atmospheric raindrop formation, where droplets coalesce within clouds until they are sufficiently large to form rain. Knowledge about the behavior of droplets during collision also aids in understanding the behavior of sprays in combustion engines or materials manufacturing, such as surface coating. Furthermore, technologies employed for solar collectors, in which a suspension is used to increase the absorption of solar radiation, also depend on droplet interactions. To optimize the corresponding technical processes, it is important to better understand the mechanisms governing droplet collision phenomena on a molecular level.

Droplet collisions have been investigated and characterized for different parameter variations, the most common of which being the Weber number^5,6^
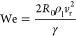
1which relates the inertia forces, embodied by the product of droplet radius *R*_0_, liquid density ρ*_l_* and squared initial relative velocity *v*_r_^2^, to the surface tension γ.^7^ Another important dimensionless property, the Reynolds number^5^
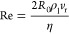
2relates the inertia forces to the viscous forces through the shear viscosity η.^8^

Laboratory experiments on droplet collisions usually consider a setup consisting of two droplet generators at a certain angle to each other, which emit droplets with a certain frequency to observe the topology of the process. Brenn et al.^9−11^ studied the behavior of droplets for a wide Weber number range and varying nondimensional impact parameter, categorizing the observed collisions into different regimes: permanent coalescence, separation of head-on or nearly head-on collisions and the formation of satellite droplets due to stretching separation. Ashgriz and Poo^1^ conducted an experimental investigation to identify two types of separation regimes for macroscale droplets, the reflexive separation regime, which occurs for head-on or near head-on collisions, and the stretching separation regime, occurring at a large nondimensional impact parameter. Qian and Law^12^ investigated water and hydrocarbon droplets in different ambient gases under varying pressure and found five regimes: bouncing, coalescence with and without notable deformations as well as stretching for head-on and off-center collisions. Willis and Orme^13,14^ conducted studies with water droplets in vacuum to eliminate the influence of the ambient gas, finding that atmospheric pressure allows for stretching of water droplets at lower Weber numbers than in a vacuum environment. Experimentally, droplet collisions have been studied extensively for a large range of the Weber number and nondimensional impact parameter.

While laboratory experiments are conducted on a macroscopic scale, collision processes occur rapidly and microscopic phenomena are excepted. Therefore, computer simulations are a powerful supplement and also offer insights into quantities which are hard to capture with experiments, e.g., highly resolved local temperature distributions. Molecular dynamics (MD) simulation is particularly suited due to its predictive power. Greenspan and Heath^15^ conducted such simulations to investigate the coalescence, separation and shattering regimes for nanoscale droplets. Wyatt^16^ used MD to study binary collisions of microscale water droplets. Ming et al.^17^ conducted one of the first systematic investigations of argon droplet collisions and also found the three regimes coalescence, separation and shattering. Svanberg et al.^18^ conducted a similar study with large water clusters. Murad and Law^19^ simulated head-on droplet collisions with MD and investigated the bouncing and coalescence regimes. Further investigations of argon droplets were conducted by Chun et al.^20^ More recent studies from Zhang et al.^21^ considered nanodroplets to investigate the bouncing and coalescence regimes, while Jiang et al.^22^ investigated the collision dynamics of nanodroplets. Moreover, Zhang and Luo^6^ and Zhang et al.^23^ studied the effects of the ambient pressure on nanodroplet collisions with MD to find the holes regime, extending the regime map of nanoscale binary droplet collisions. They found how expected regime outcomes change as the droplet size goes from the nanoscale toward the macroscale (i.e., how the probability of the holes regime decreases with rising droplet size). Further, MD was used by Liu et al.^24^ and Wang et al.^25^ to study the coalescence of nanodroplets for a wide range of Weber and Ohnesorge numbers, varying the nondimensional impact parameter, considering coalescence, stretching separation and shattering.

This work focuses on collisions of droplets which are in equilibrium with their surrounding vapor. In addition, the present systems involve a much larger number of molecules (up to 2 × 10^7^) compared to previous studies. By simulating collisions with such a high molecular count, this study explores collision dynamics involving large droplet radii, which might help to bridge the gap between the nano- and the macroscale. Compared to other work in the literature, which usually considers only one or two radii, this work also investigates the influence of droplet size through four different radii ranging from 30 to 120 molecule diameters. By using such large droplet radii, it is possible to attempt to investigate the differences between the micro- and the macroscale behavior during collision. Also unique is the wide initial relative velocity range simulated for each radius. The droplets in the present work are assigned with their true saturated liquid density and placed in a vapor environment with the corresponding saturated vapor density to avoid superimposed evaporation processes. Moreover, it goes beyond analyzing collision topology by offering a more comprehensive analysis of collision dynamics. Based on spatially resolved density, velocity and temperature distributions throughout the collision process, deeper insights into various physical properties are gained. E.g., the spatially resolved density allows for the quantitative analysis of the collision topology that is often accompanied by the formation of a disc with a rim. Moreover, the region where the kinetic impact energy is primarily converted to thermal energy is clearly identified.

## Methods

**Figure 1:**
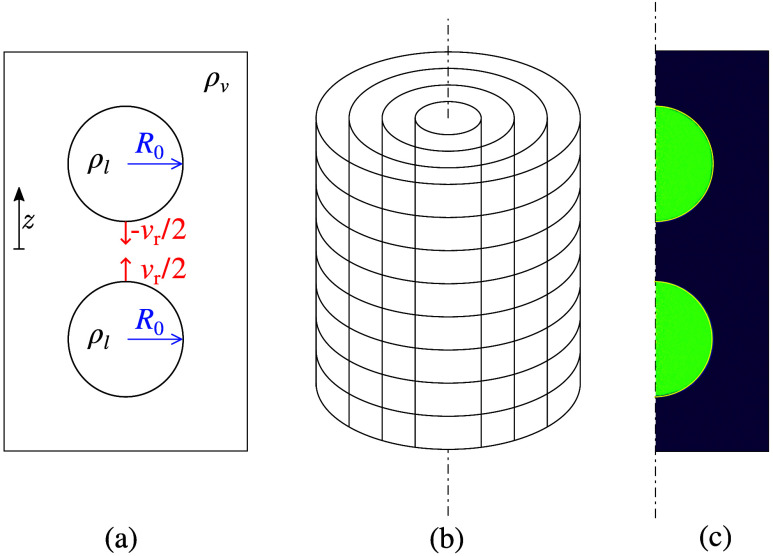
Schematic of the initial setup of head-on nanoscale droplet collisions (a) and the cylindrical sampling grid (b) used to sample the relevant volume. Due to rotational symmetry, the simulation data were averaged onto a plane (c).

This work considers head-on^26^ nanoscale^6^ droplet collisions as outlined in Figure 1. To better categorize the 36 collision cases studied in this paper, different criteria, like Weber number and topological collision result, are summarized in Table 1, which were used for the classification of the outcomes into separate regimes.

**Table 1:** Classification of the Collision Regimes

regime	topology	Weber number	result
bouncing^6,13,26^	droplet sides facing each other flatten during approach; build-up of ambient gas pushes droplets away from each other	very low (∼10^0^)	two separate droplets
coalescence^27−29^	droplets merge with little to no deformations during collision	low (∼10^1^)	one stable droplet
stable collision^26^	formation of a disc during collision	low to moderate (∼10^2^)	one stable droplet
holes^6^	formation of a disc with a rim during collision; formation of holes in the disc; formation of a torus-like geometry before droplet formation	high (∼10^3^)	one stable droplet
shattering^6^	formation of a disc with a rim during collision; formation of holes in the disc; breakup of the disc and the rim	very high (∼10^3^)	many satellite droplets

The Lennard-Jones (LJ) potential^30,31^ was employed to describe the intermolecular interactions^32^
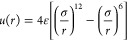
3with *r* being the distance between the two molecules, ε the energy parameter and σ the size parameter,^27^ which roughly coincides with the molecule diameter. The first term ∼*r*^–12^ represents the repulsive forces due to Pauli exclusion, whereas the second term ∼*r*^–6^ considers the attractive forces due to dispersion.^33^

The truncated and shifted Lennard-Jones (LJTS) fluid is a form of that model in which the potential energy is truncated and shifted to zero at a certain cutoff radius *r*_c_
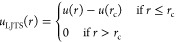
4meaning that for molecule pairs with a distance beyond the cutoff radius, the potential energy is set to zero.^34^ This work used a cutoff radius of *r*_c_ = 2.5 σ. Due to the truncation, no long-range corrections need to be considered, which is particularly beneficial for inhomogeneous systems that undergo topology changes.^35^ The LJTS potential is nonetheless a good choice as it properly describes the thermodynamic properties of simple fluids, such as the noble gases and methane.^36,37^ Moreover, thermodynamic properties and equations of state are well established for this model fluid. When working with LJ fluids, all physical properties are usually reduced using a dimensional analysis based on the potential parameters, cf. Table 2. Most results are presented here in this reduced form, omitting the asterisk that is often used to indicate this reduction. Consequently, the results are valid for any combination of σ, ε and *m*, including the specific model parameters of argon,^36^ with σ = 0.33916 nm, ε/*k*_B_ = 137.90 K and *m* = 39.948 g/mol, where *k*_B_ denotes the Boltzmann constant. Note that for argon, a time interval in reduced units Δ*t* = 1 corresponds to about 2 ps.

**Table 2:** Physical Properties in Reduced Units^34,38,39^

property	reduced	SI unit
length	z* = *z*/σ	m
time		s
velocity		m/s
temperature	T* = *Tk*_B_/ε	K
density (molar)	ρ* = ρσ^3^*N*_A_	mol/m^3^
pressure	p* = *p*σ^3^/ε	N/m^2^
surface tension	γ* = γσ^2^/ε	N/m
shear viscosity		N·s/m^2^
thermal conductivity		W/(m·K)

The density of the saturated liquid ρ_l_ constituting the droplets and its coexisting vapor ρ_v_ were specified according to Vrabec et al.^34^ At the temperature under investigation, the density ρ_l_ of very small droplets is up to 0.6% larger than the saturated liquid density of a bulk phase ρ′ and the vapor density ρ_v_ is also by up to 12% larger than the saturated vapor density of the bulk ρ″ due to the small droplet radius *R*_0_. This is rationalized by the Laplace equation
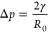
5where Δ*p* is the pressure difference between the coexisting phases due to the surface tension acting on a (strongly) curved interface. In this study, colliding droplets and the surrounding vapor phase were prepared such that it was accounted for the increased vapor pressure.^40^

The surface tension was calculated with a function that was fitted to data presented by Vrabec et al.^34^ Therein, it was sampled via the Irwing–Kirkwood pressure tensor for varying temperature and droplet size, as the surface tension is a function of these parameters. The fit created in this work for the surface tension on the basis of data by Vrabec et al. is given by
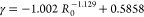
6

**Figure 2:**
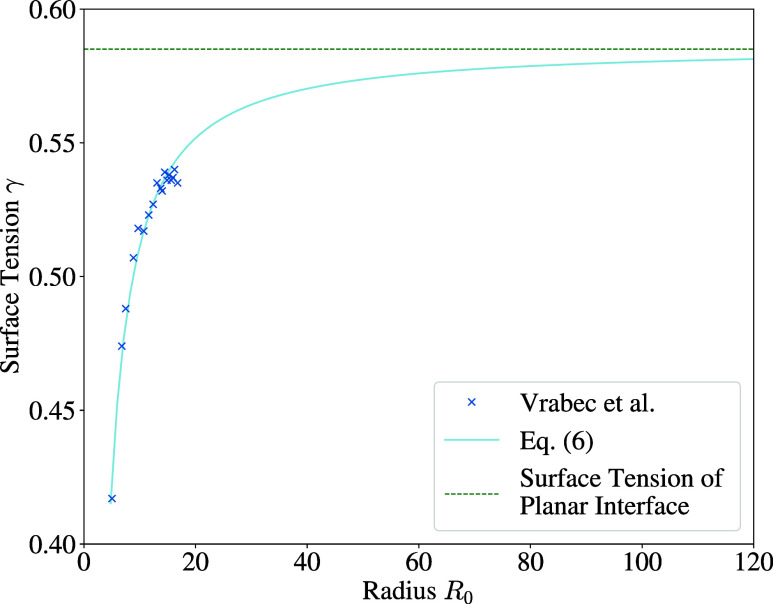
Surface tension γ at *T* = 0.7 as a function of droplet radius *R*_0_ based on data from Vrabec et al.,^34^ considering the planar surface tension at *R*_0_ → ∞.

Due to the fact that this work considered only droplets with an initial temperature of *T* = 0.7, the surface tension in eq 6 is only a function of the radius. The macroscopic limit *R*_0_ → ∞ was included in the fit, representing the surface tension of the planar interface, cf. Figure 2.

The shear viscosity^41^ and the thermal conductivity^42^ of the LJTS fluid were calculated according to Lautenschläger et al.^43^ The speed of sound and the isochoric heat capacity were determined with the perturbed truncated and shifted (PeTS) equation of state.^44^

The present work used the eccentricity to determine the deformation of the colliding droplets, which is given as
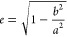
7where *a* is the length of the semimajor axis from the center and *b* is the length of the semiminor axis from the center. A sphere is characterized by *e* = 0 and a disc by 0 < *e* < 1. The closer *e* gets to unity, the flatter the disc is.^45^

As the droplets collide, their size in radial direction *a* increases, while it decreases in axial direction *b*. When calculating the eccentricity at the point of maximum deformation, conclusions can be drawn with respect to the topology of the colliding droplets.

For collisions in the coalescence regime, it is expected that the eccentricity remains close to zero, since the inertia forces are small compared to the surface tension, which limits their deformation due to inertia forces. The colliding droplets remain rather spherical and hence have an eccentricity close to zero. For stable collisions at a higher Weber number and in the holes regime, the droplets’ inertia increases, creating a disc-like structure. The thinner this disc becomes, the eccentricity approaches unity and the more unstable the resulting droplet becomes during collision. This is due to its increasing maximum extension in radial direction, which eventually reaches a point where the surface tension cannot balance the inertia forces. For collisions in the shattering regime, the eccentricity looses its interpretability, since the colliding droplets completely disintegrate. Therefore, it was not used for the shattering regime.

In addition to the eccentricity, a scaling law was used to quantify the maximum deformation of the colliding droplets. It is given by^46^
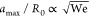
8where *a*_max_ is the maximum radius of the disc.

The parallelized MD^47^ code *ls1 mardyn*(48) was used to simulate the influence of droplet radius and initial relative velocity on the droplet dynamics during collision at a constant temperature *T* = 0.7 and ambient pressure of *p* ≈ 0.005. The Prandtl number^49^ Pr ≈ 0.83 was almost constant for all cases. Table 3 lists all 36 droplet collision cases investigated in this work. To get an idea of the magnitude of the velocities, they can be compared to the speed of sound which is in reduced units *c*_v_ ≈ 1.05 in the vapor and *c*_l_ ≈ 4.9 in the liquid under the present conditions. The software *MegaMol*(50,51) was used to visualize and categorize the collision processes into regimes based on their topology according to the criteria in Table 1.

**Table 3:** Droplet Collision Cases Investigated in the Present Work

	initial relative velocity *v*_r_										
radius *R*_0_	0.25	0.5	0.75	1.0	1.25	1.5	1.75	2.0	2.5	3.0	3.5
30	x	x	x	x	x	x	x	x	x	x	x
60	x	x	x	x	x	x	x	x	x	x	
90	x	x	x	x	x	x	x	x	x		
120		x		x	x	x	x	x			

To obtain detailed information on the process, a sampling tool was used to measure temperature, density and hydrodynamic velocity during collision by dividing the domain into cylindrical shells, cf. Figure 1. By plotting the sampled property profiles in a two-dimensional form, the collision outcomes were further analyzed. Due to rotationally symmetric decomposition and averaging, the quantities were sampled in cylindrical coordinates. Care has to be taken concerning the temperature. Since the temperature is associated exclusively with the thermal, i.e., undirected, velocity of the molecules, the local hydrodynamic velocity must be subtracted from each molecule’s individual velocity. In cylindrical coordinates, the hydrodynamic velocity components of an ensemble of *N* molecules are given by
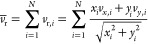
9
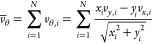
10
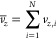
11with the velocity vector of molecule *i* in Cartesian (*v*_*x*,*i*_, *v*_*y*,*i*_, *v*_*z*,*i*_) and cylindrical coordinates (*v*_r,*i*_, *v*_θ,*i*_, *v*_*z*,*i*_). The Cartesian distances *x*_*i*_ and *y*_*i*_ of the respective molecule *i* are measured from the rotation axis. Based on these velocities, the temperature can then be calculated by^52^
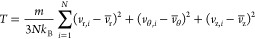
12for the present monatomic molecules.

## Results and Discussion

For each of the cases simulated in this work, the collision outcome was analyzed based on the topology seen in the visualization software *MegaMol*. Table 4 lists the conditions under which these collisions took place.

**Table 4:** Parameters and Outcome of the Collision Cases Simulated in the Present Work

*R*_0_	*v*_r_	We	Re	regime
30	0.25	5.26	5.34	coalescence
30	0.5	21.0	10.7	stable collision
30	0.75	47.3	16.0	
30	1.0	84.1	21.4	
30	1.25	131.4	26.7	
30	1.5	189.3	32.1	
30	2.0	257.6	37.4	
30	2.5	336.5	42.7	
60	0.25	10.3	10.8	
60	0.5	41.1	21.6	
60	0.75	92.5	32.5	
60	1.0	164.7	43.3	
60	1.25	256.8	54.1	
60	1.5	369.9	64.9	
60	1.75	503.4	75.7	
90	0.25	15.3	16.3	
90	0.5	61.2	32.5	
90	0.75	137.7	48.9	
90	1.0	244.8	65.2	
90	1.25	382.5	81.5	
120	0.5	81.3	43.5	
120	1.0	325.3	87.1	
120	1.25	508.2	108.8	
60	2.0	657.5	86.5	holes
90	1.5	550.8	97.8	
90	1.75	749.7	114.1	
120	1.5	731.9	130.6	
30	3.0	757.1	64.1	shattering
30	3.5	1031	74.8	
60	2.5	1027	108.2	
60	3.0	1479	129.8	
90	2.0	979.2	130.3	
90	2.5	1530	162.9	
120	2.0	1301	174.2	

It has been shown in the literature that the bouncing regime occurs for nanoscale droplets at a very low Weber number if at least one additional gas species with sufficient ambient pressure is present.^6,53^ The bouncing regime was not encountered in this work due to the presence of a pure fluid in the entire simulation domain.^54^

This work observed one case in the coalescence regime, which is characterized by the merger of two droplets with low kinetic energy and is associated with weak deformations during the collision process, leading to one stable droplet. Even with the final outcome being the same as in the stable collision regime, this work still differentiates between the two due to the topology difference during collision.

**Figure 3:**
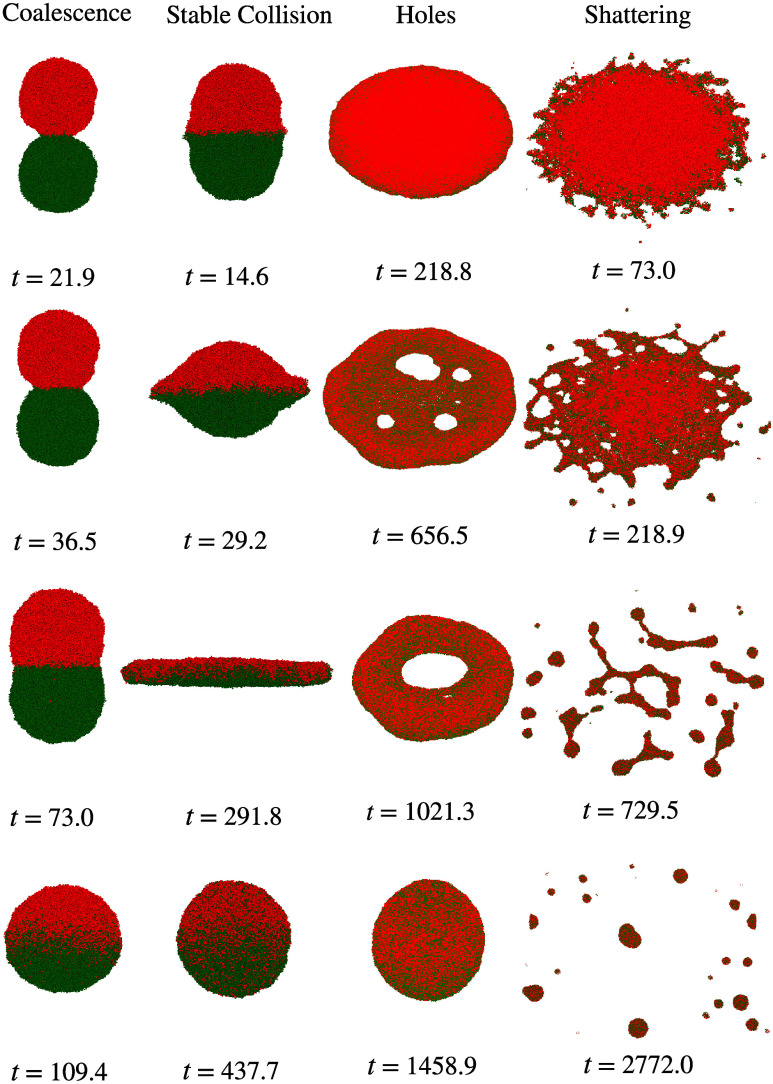
Topology of the four observed regimes over time: coalescence (far left), stable collision (center left), holes (center right) and shattering (far right). Note that the time origin *t* = 0 was set to the moment of contact and time proceeds from top to bottom. The shown cases are Coalescence *R*_0_ = 30, *v*_r_ = 0.25; Stable collision *R*_0_ = 30, *v*_r_ = 1.75; holes *R*_0_ = 60, *v*_r_ = 2.0 and shattering *R*_0_ = 90, *v*_r_ = 2.5.

Figure 3 shows snapshots of the progression in the coalescence regime. In these visualizations, all molecules that were initially part of the vapor phase as well as molecules diffusing into the vapor were graphically taken out so that the droplets can be seen more clearly. Of course, molecules constituting the vapor phase also diffuse into the droplets, but both processes are not visible in Figure 3 because the relevant molecules were graphically taken out. The defining topology of coalescence is a lack of significant deformations, as two droplets simply merge. The change of length in radial direction shows this as well. Because no disc-like structure forms during collision, no true maximum extension in radial direction is attained, which can also be seen in terms of eccentricity for this collision case.

Since we studied coalescence with MD in much detail in preceding work,^27^ accompanied by phase-field CFD modeling, the focus was laid here on the other regimes.

This work categorized events into the stable collision regime based on the formation of a disc-like structure during collision, without any holes or tears therein, and the emergence of a single, stable droplet at the end of the process. This regime has to be differentiated from the coalescence regime as it occurs at higher velocities and larger droplet radii, which are associated with larger kinetic energies and greater deformations during the collision process. Table 4 lists the 24 collisions sampled in this work that were categorized into this regime.

Figure 3 depicts the progression of an exemplary collision case in this regime to show the deformations that can occur. At the beginning, the droplets form a bridge in between them, but they do not simply glide into each other as in the coalescence regime. For the depicted case at the time instance *t* = 14.6 and later, it can be observed that the merging object stretches out in radial direction, creating a rim sticking out and stretching the droplet into a disc with a rim at *t* = 29.2, for example. Once the expansion of the disc reaches its maximum, the droplet retracts due to surface tension, eventually stabilizing into one unit, as seen at *t* = 291.8 and 437.7.

The holes regime was observed in this study for all droplet radii, except for the smallest *R*_0_ = 30, which may be too small to enter the holes regime. Other studies of nanoscale droplets, such as by Kalweit and Drikakis,^38^ have also not observed the holes regime. It is important to note that the droplets simulated in their study^38^ were much smaller than the ones considered in this work, but they concluded that the holes regime only appears above a certain droplet radius threshold. Table 4 lists the four cases in which the holes regime was observed here.

Figure 3 shows the topology of a typical case in the holes regime. Here, an angular view from the top was chosen to depict the holes formed in the planar structure. In this regime, the initial stage is similar to the stable collision regime, since a disc-like structure is formed. However, the spread in radial direction is greater than in the stable collision regime, leading to an even thinner disc with a rim. This very thin disc with a rim then tears, leading to the formation of holes in the disc as surface tension forces retract the droplet. As this continues, the holes grow and merge into a single hole within a torus-like structure, as seen at *t* = 1021.3. Surface tension forces proceed to dominate so that this torus-like structure merges into one stable droplet at *t* = 1458.9.

The shattering regime was observed in this work in seven cases encompassing all droplet radii, cf. Table 4. Figure 3 shows the two droplets colliding, with deformations occurring early on in the collision process. The disc-like structure that is formed disintegrates at its edges, a process supported by the high temperature in this region, with small holes forming therein, as opposed to the disc-like structure formed in the holes regime, which appears more as a disc with smooth edges. Later on, at *t* = 218.3, the formation of holes can be seen along the outer edges of the disc as well as in the center. The disc then ruptures and disperses into many satellite droplets.

**Figure 4:**
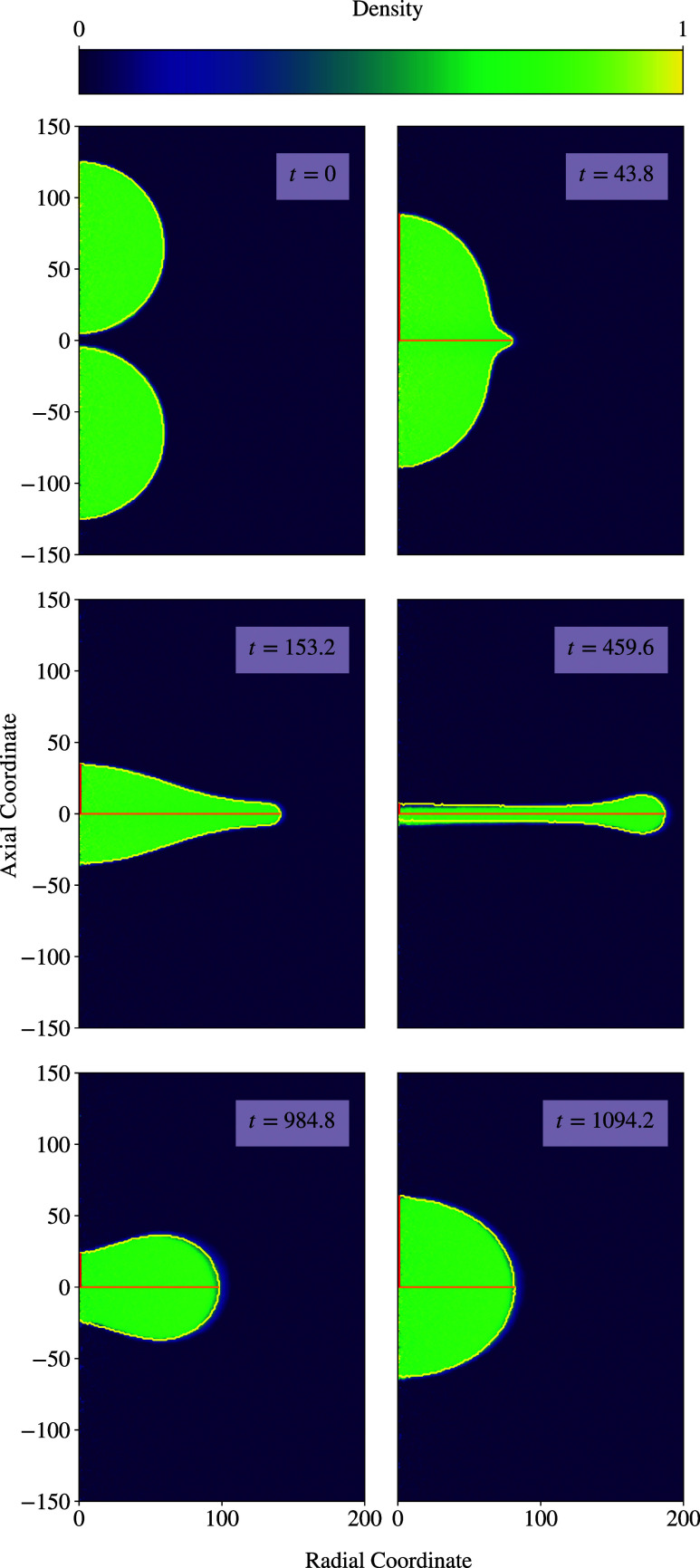
Rotationally averaged density profile of the collision case *R*_0_ = 60 and *v*_r_ = 1.75. The yellow curve indicates the interface, while the straight lines show the semimajor axis *a* (orange) and the semiminor axis *b* (red).

Using the rotationally averaged cylindrical sampling data, the density profile was employed to measure the deformation of the droplets during collision by examining the variation of its axes lengths in radial and axial directions. Figure 4 depicts the progression of a density profile. Here, the outline of the droplet’s interface was detected by a threshold based on the density condition ρ ≈ ρ_*l*_, identifying contours in the data, the largest of which representing the droplet interface. The area within this contour was then used to measure the change of length in radial direction and axial length from the top edge of the droplet to the center.

**Figure 5:**
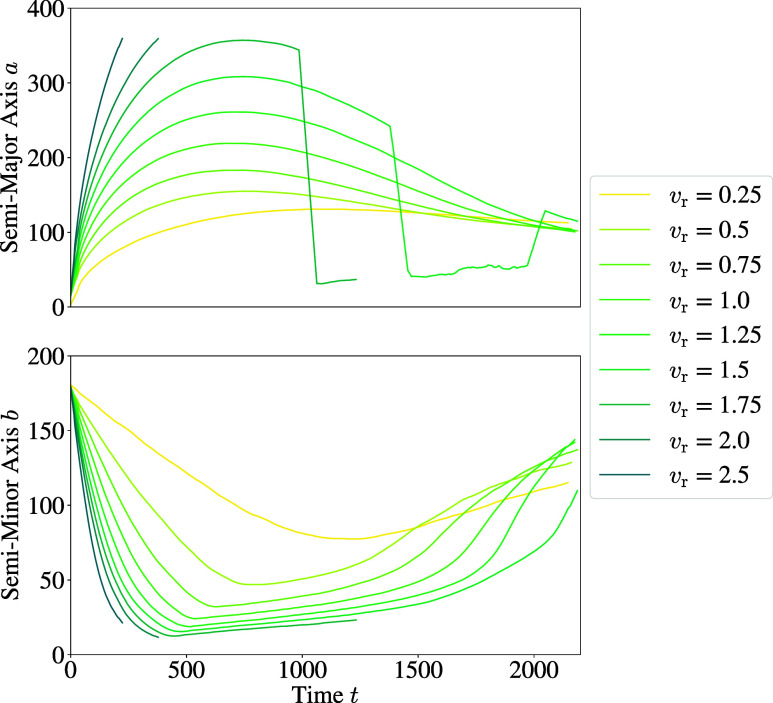
Plots depicting the change in the length of the semimajor axis (top) and the semiminor axis (bottom) for the *R*_0_ = 90 droplets with a varying initial relative velocity *v*_r_.

These lengths were then plotted for all collision cases for a given droplet radius so that the collisions can also be classified according to the change in axes lengths, cf. Figure 5.

Figure 5 (top) shows the change in radial axis length. Since the measurements started at the moment of contact, the initial radial axis length was zero. Then the colliding droplets begin to merge, leading to an increase in radial length. In the coalescence regime observed in this work, the droplets simply merge to become one unit so that the curve monotonously increases to converge toward the maximum expansion, which can be seen for *R*_0_ = 30 in Figure A6 in the Supporting Information (SI). The curve then stagnates, showing a clear difference compared to the change in radial direction of collisions in the stable collision regime. For stable collisions, a disc with a rim is formed during collision, entailing an increase in length in radial direction that peaks and then reclines to converge against a constant. The maximum of this curve depends on the Weber and Reynolds numbers. The holes regime can be classified by a sudden drop in length in radial direction. The droplets first expand into a disc with a rim, then form holes within the disc, which leads to the torus-like structure seen in Figure 3. Once the ring retracts to form a droplet, the length in radial direction suddenly increases again. The shattering collision can be identified by the sudden stop early on in the collision process since satellite droplets break off here.

Figure 5 (bottom) depicts the change of length in axial direction. Since these plots show the progression for the *R*_0_ = 90 droplet, the lengths start at 180 (the moment when the droplets meet). As the droplets merge, becoming more and more disc-like, the axial length falls. If the droplets form a stable droplet at the end, the length in axial direction rises and converges toward a constant value. If the droplets shattered, the lengths were not recorded after disintegration.

**Figure 6:**
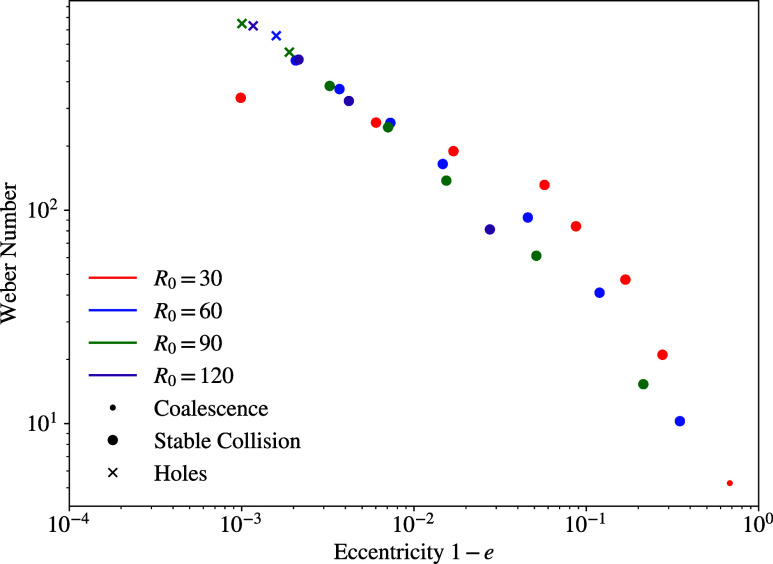
Weber number over 1 – *e*, where *e* is the eccentricity.

For each droplet collision, the eccentricity was calculated by taking the maximum value of the radial length and its corresponding axial length value. This allows for the eccentricity to be measured at the maximum extension in radial direction when the disc with a rim is most pronounced. The closer the eccentricity value is to unity, the flatter the formed disc. Figure 6 depicts the Weber number over eccentricity. It shows that the closer the eccentricity is to unity (to the left in Figure 6), the more unstable the collisions become since the holes regime occurs at *e* → 1. For the stable collision regime, it can be seen that the higher the relative velocity, and therefore the Weber number, the wider the disc spreads, which promotes the instability of the collision. Moreover, the only collision case where *e* → 0 was observed is the coalescence case, which shows that even at maximum extension, the topology of coalescing droplets is still close to that of a sphere, which differentiates this collision from the stable collision cases.

**Figure 7:**
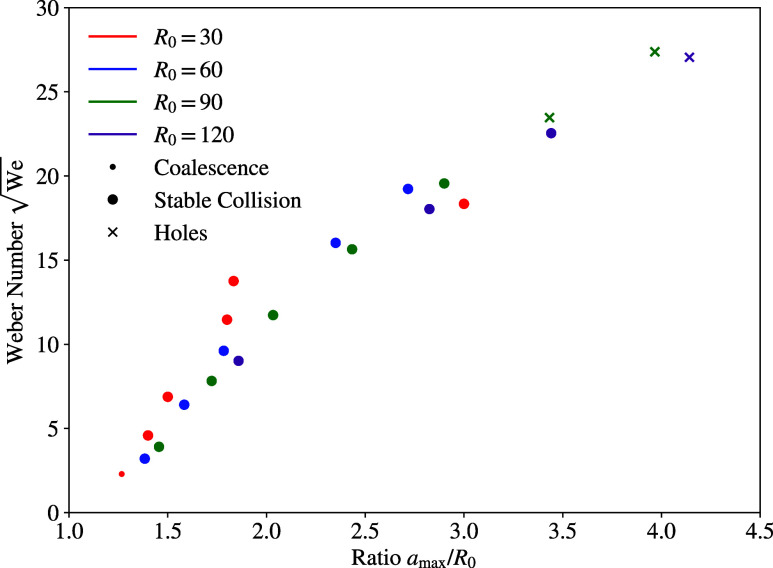
Square root of the Weber number over the radius ratio *a*_max_/*R*_0_.

Figure 7 shows the square root of the Weber number over the ratio of maximum disc deformation and initial radius. For the cases in the coalescence and stable collision regimes, an almost linear proportionality can be observed, which is largely independent on the initial radius. In case of the holes regime, the measured maximum deformation is slightly too large to match this linear relation. This is directly connected to the formation of holes, which allows the disc to spread wider.

**Figure 8:**
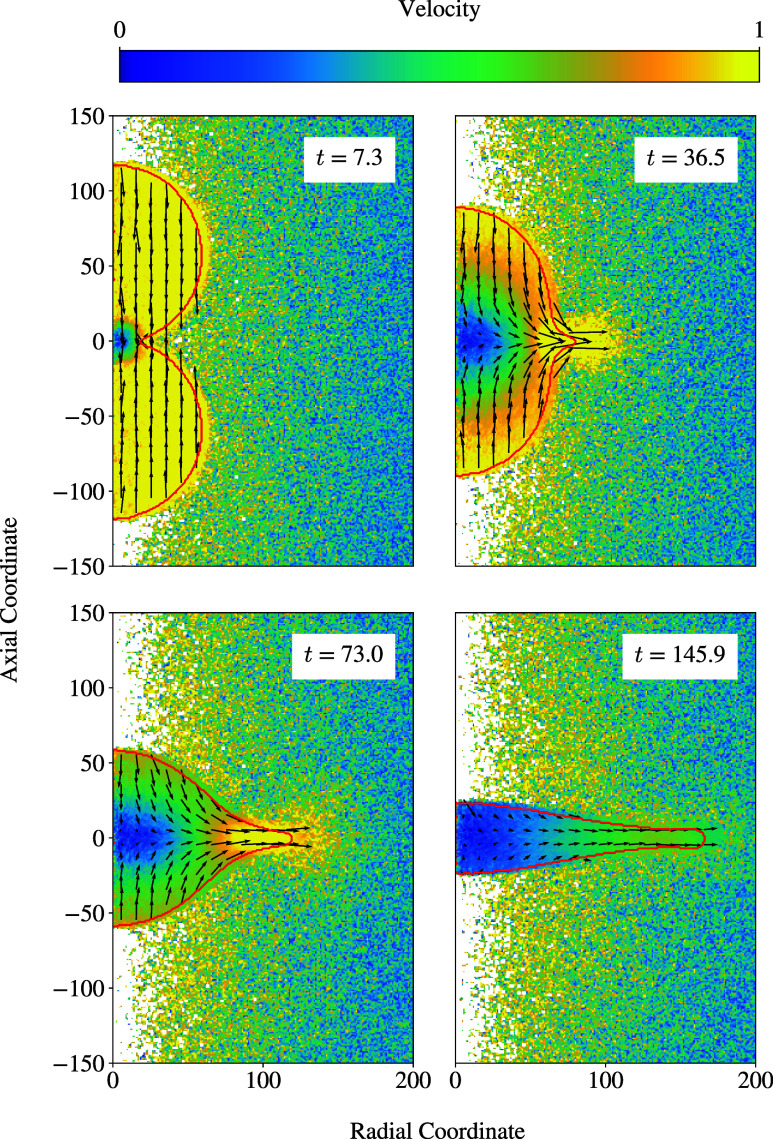
Hydrodynamic velocity profiles of the collision case with *R*_0_ = 60 and *v*_r_ = 2.0 over time. The arrows indicate the direction and magnitude of the local hydrodynamic velocity.

Figure 8 shows velocity profiles during an exemplary droplet collision. The vectors depict the local hydrodynamic velocity, showing that at the beginning, the liquid propagates with the initially assigned relative velocity, and then stalls in the center of the bridge formed during collision.

It becomes apparent that the liquid spreads out radially with the initial relative velocity, until coming close to the point of maximum expansion. At time instance *t* = 36.5, for example, the center of the formed droplet has a velocity close to zero, but the outskirts still have a velocity close to the initial relative velocity as the colliding droplets flow outward to form the disc.

When examining droplet collisions for a given droplet radius *R*_0_, the liquid density ρ_*l*_ and surface tension γ remain fairly constant, leading to the initial relative velocity *v*_r_ being the only influence on the Weber number. For example, when looking at droplet collisions with *R*_0_ = 60, it can be seen that for low relative velocities, the droplets form a stable droplet and are part of the stable collision regime. As the initial relative velocity increases, and with it the Weber number, the holes regime starts to dominate, before moving on into the shattering regime. Therefore, as expected, the higher the initial relative velocity, and thus the Weber number, the more unstable a collision becomes.

**Figure 9:**
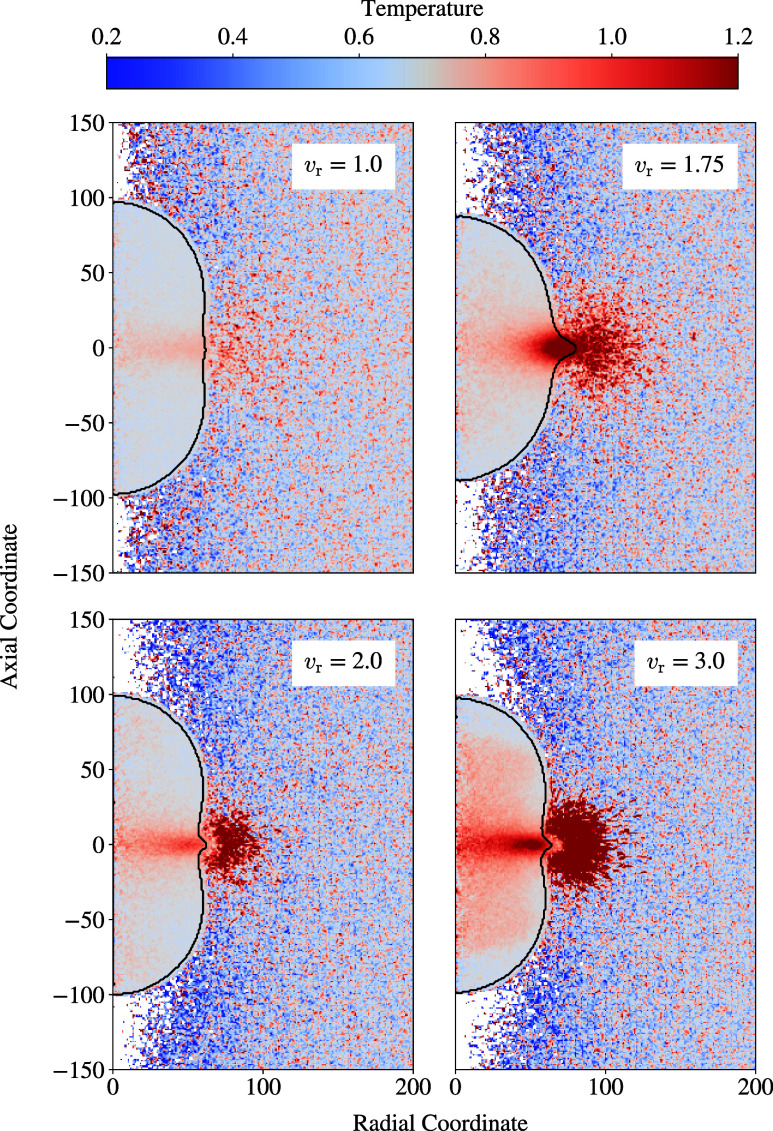
Temperature profiles during the collision of droplets with *R*_0_ = 60 for varying initial relative velocity *v*_r_.

The initial relative velocity has an impact not only on the topology, but also on other phenomena that occur during collision, such as the conversion of kinetic energy into thermal energy.^55,56^Figure 9 compares droplet collision cases with varying *v*_r_ with respect to this aspect. Temperature profiles are depicted at the same length in radial direction *a* since this indicates a comparable instantaneous topology during the collision for different *v*_r_.

Figure 9 shows that the higher the initial relative velocity, the more kinetic energy is converted into thermal energy during the collision process.^56,57^ As the droplets collide, repulsive molecule interactions dominate in the bridge along the axial coordinate, which pushes the droplet outward in radial direction. Primarily at the radial edge, the conversion of kinetic energy into thermal energy occurs. In addition to this conversion, a part of the kinetic energy is also transformed to surface energy due to the increasing interface area during collision.

As expected, from the Weber number it can be seen that also the radius has an influence on the collision process. For example, at constant initial relative velocity *v*_r_ = 2.0, the resulting collision regime varies with the radius. For a small radius *R*_0_ = 30, the stable collision regime dominates. However, with increasing *R*_0_ at a constant initial relative velocity, first the holes regime and then the shattering regime prevails.

**Figure 10:**
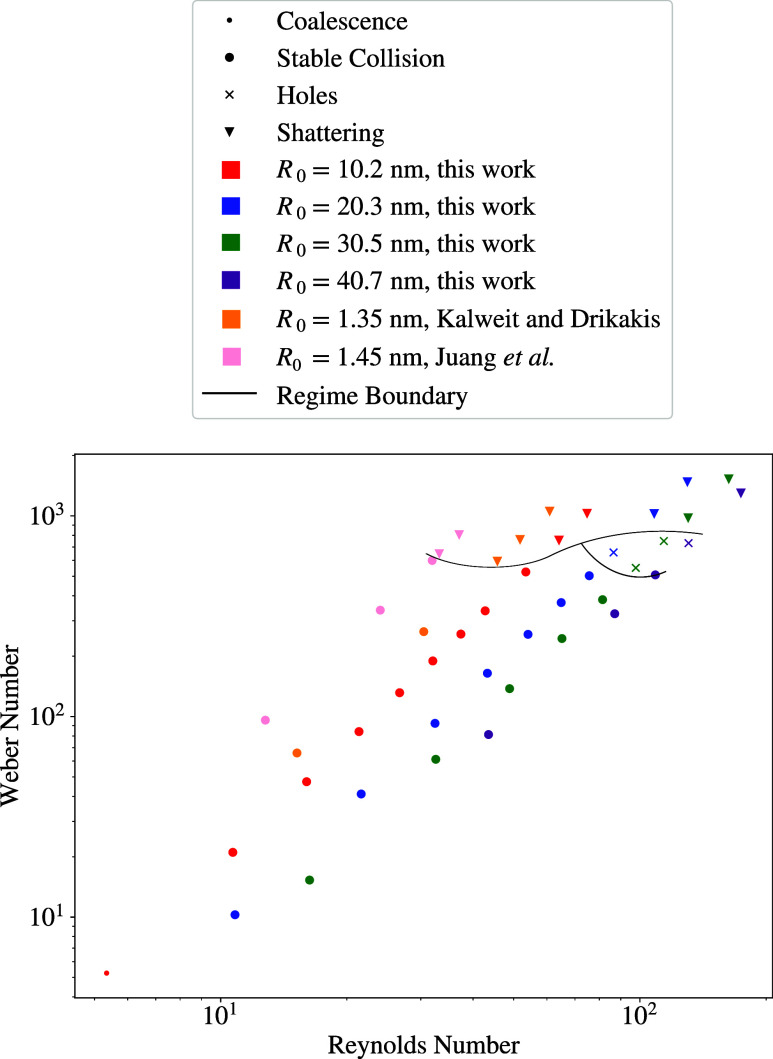
Weber number over Reynolds number and estimated regime boundaries based on the present work and literature data by Kalweit and Drikakis^38^ as well as Juang et al.^58^ For this comparison, the present data were converted to SI units using the potential parameters for argon.

The collision cases simulated in this work allow for conclusions on the regime ranges so that other collision scenarios can be anticipated. Figure 10 shows the collision regimes and their boundaries that were observed in this work. Note that the estimates for the collision outcome that can be made from this work are only valid within the ranges of the varied parameters, while the present initial droplet radius *R*_0_ was converted to SI units assuming potential parameters for argon.

Figure 10 shows the regime boundaries that were concluded from the present simulation results. The coalescence regime is treated as a part of the stable collision regime, occurring at very small velocities, before moving on to the stable collision regime with rising Weber and Reynolds numbers. The holes regime dominates for droplet radii of *R*_0_ = 60, 90, and 120. The regime boundary lines are assumed to bend, as this regime can only be observed for nanoscale droplets. Thus, it can be assumed that with increasing droplet radius, the possibility for this regime decreases. Furthermore, the collision case *R*_0_ = 120 and *v*_r_ = 1.25 seems to be very close to this regime boundary, since when observing its topology in detail, it became evident that tiny holes did form during the collision and closed rapidly before being able to develop and spread. The shattering regime then dominates at high Weber and Reynolds numbers. Table 5 shows the ranges in which this work estimates the separate regimes to be for the LJTS fluid in the investigated parameter range.

**Table 5:** Comparison of Estimated Regime Ranges with the Literature

	this work	Zhang and Luo^6^	Zhang et al.^23^
droplet fluid	argon	water	water
droplet radius [nm]	10.2, 20.3, 30.5, 40.7	10	10
ambient pressure [bar]	∼0.69	∼2.7	0
Collision Regimes: Weber Number Ranges			
coalescence	≲10		
stable collision	∼10–505	≲ 530	277 (one case)
holes	∼505–750	∼530–667	426 (one case)
shattering	≳750	≳667	540 (one case)

First, it should be noted that the type of liquid, temperature and ambient pressure under which collisions occur influence the collision regime boundaries so that a direct comparison with other findings is not always straightforward. The results of this work are compared with literature data in Figure 10, where two other MD studies using argon are included. This was done by converting the reduced units used in those studies to reduced units comparable to this study. Kalweit and Drikakis^38^ studied droplet collisions with MD simulations using a 12–6 LJ potential by varying the initial relative velocity and impact parameter. For comparison, only their findings with an impact parameter of zero were used, since this work focuses on head-on collisions. For the radius *R*_0_ = 1.35 nm used in their work, they found that the coalescence/stable collision regime dominates until *v*_r_ ≈ 5.02 and We ≈ 802.79, leaving the final collision case examined at *v*_r_ ≈ 5.9 and We ≈ 1108.93 in the shattering regime. Note that no holes regime was found by them. A similar study by Juang et al.^58^ investigated head-on collisions of argon droplets, also using a 12–6 LJ potential. Here, a wider Weber number range was considered, with coalescence/stable collision regimes being observed up to a Weber number of 482.30, while the shattering regime was observed at We = 600.78 and 753.61. Furthermore, it should be noted that the ambient pressure under which these collisions occur affects the collision regime. With higher system pressure, the coalescence/stable collision regimes occur at higher Weber numbers, since a larger ambient pressure acts as a cushion. Therefore, only collision cases from Juang et al. under an ambient pressure of *p* = 0.56 bar (close to the ambient pressure of *p* ≈ 0.69 bar in this work) are compared.

The collisions investigated by Kalweit and Drikakis as well as Juang et al. considered a smaller initial droplet radius than this work. As discussed, it has been found that smaller droplet radii lead to a larger stable collision regime, so that the shattering regime occurs at a higher Weber number than with larger droplet radii. Since both Kalweit and Drikakis as well as Juang et al. simulated droplets with much smaller radii, this could be the factor that led to the stable collision regime occurring up to higher Weber numbers than the collision cases simulated in the present work. This, along with the consideration that different fluids or ambient pressures were used, could explain the discrepancies, with the coalescence/stable collision regime occurring at a much higher Weber number. Nonetheless, the literature studies support the assumption that the droplet radius impacts collision dynamics by decreasing the boundary of the stable collision regime with rising droplet radius. Furthermore, the general trend observed in this work with increasing initial relative velocity leading to more unstable collisions was also found by Kalweit and Drikakis as well as Juang et al.

Another fluid that has often been used to simulate droplet collisions is water, which is not as straightforwardly comparable through a conversion of reduced units. However, the findings of these works still relate to the trends found here, cf. Table 5.

Zhang and Luo^6^ used MD to simulate water droplets in a nitrogen gas ambient. The droplets had an initial radius of 10 nm and droplet collisions for a Weber number range were simulated at 0, 2.7, and 8 bar. Since the ambient pressure has an influence on the collision regime boundaries, with a higher pressure resulting in stable collisions for higher Weber numbers, the findings of Zhang and Luo for an ambient pressure of 2.7 bar (roughly 4 times the ambient pressure of 0.68 bar used in this work) are discussed here. Zhang and Luo found that the coalescence/stable collision regime occurs until about a Weber number of 530 for water droplets in nitrogen gas ambient and that the holes regime occurs between a Weber number of ∼530 to 667. This work found the stable collision regime to occur up to a Weber number of 505, which is somewhat below that of Zhang and Luo. This could be due to the higher ambient pressure of Zhang and Luo, which acts as a cushion to prevent early holes formation or shattering during collision. However, especially when comparing the shattering regime, which occurs at Weber numbers slightly smaller than the findings of this work, it is likely that earlier shattering is due to the difference in fluid properties.

Zhang et al.^23^ conducted a study simulating water droplets in vacuum, with an initial droplet radius of 10, 50, and 100 nm. Since this work simulated droplet radii of up to 40.7 nm, the findings for their 50 nm droplet radius are discussed here. Zhang et al. did not specify regime boundaries in their work, but discussed characteristic collisions for each regime. The characteristic collisions found in their study, the stable collision regime at We = 277 and the holes regime at We = 500, occur at slightly lower Weber numbers than the regime boundaries identified in this work, but remain largely within the present regime boundaries. This correlates with the assumption that the difference in ambient gas allows for the holes and shattering regime to occur at different Weber numbers and that the fluid properties of water lead to slightly different collision dynamics. Another important finding of Zhang et al. is the transition from nanoscale to macroscale droplet collisions. As discussed above, nanoscale droplet collisions present five main regimes, i.e., bouncing, coalescence, stable collision, holes and shattering. By simulating larger droplets at 50 and 100 nm, Zhang et al.^23^ were able to find the transition in the expected regimes for larger droplets.

A transition from the holes regime to a reflexive separation regime occurs when the droplet radius increases.^23^ Zhang et al. found that for a droplet radius of 50 nm, the holes regime was still observed. However, when simulating droplets with an initial radius of 100 nm, a reflexive separation regime occurred at Weber numbers where the holes regime is expected. This correlates with the estimations made in this work, that with higher droplet radius the occurrence of the holes regime decreases, as can be seen in the regime boundaries drawn in Figure 10. Therefore, it can be said that the regime ranges in this work are confirmed by other findings, considering the discrepancies of fluid properties, ambient pressure and droplet radius investigated.

## Conclusions

This work employed large molecular dynamics simulations to investigate binary droplet collisions of nanoscale droplets consisting of 0.33, 2.5, 8.3, and 20 × 10^6^ molecules interacting via the 12–6 LJTS potential. Head-on collisions were simulated with a constant nondimensional impact parameter of *X* = 0 at four different radii of *R*_0_ = 30, 60, 90, and 120 molecule diameters, which might help to bridge the gap to experiments due to the comparably large size of the droplets. For each droplet radius, collisions were sampled for 11 initial relative collision velocities of *v*_r_ = 0.25 to 3.5, except for the *R*_0_ = 120 droplet, where seven collision cases were considered, covering a somewhat smaller initial relative velocity range.

Of the five regimes that are known for head-on nanodroplet collisions (bouncing, coalescence, stable collision, holes and shattering), this work observed the latter four. The coalescence regime was found to occur at very low Weber and Reynolds numbers (collision case *R*_0_ = 30 and *v*_r_ = 0.25). The stable collision regime that results, like coalescence, in one stable droplet, but with significant deformations during the collision process, was found to occur within a Weber number range of approximately 10 to 505. Furthermore, the holes regime was observed for droplets of *R*_0_ = 60, 90, and 120 in a Weber number range of approximately 505 to 750. Collision cases with a higher Weber number were categorized into the shattering regime, where many satellite droplets form.

This work found that for the LJTS fluid at the simulated temperature conditions, the holes regime occurred for a droplet radius of *R*_0_ = 60, 90, and 120. It was also observed that the probability of the holes regime expands first with a larger radius, and then declines, since for the largest radius *R*_0_ = 120 a stable collision case was observed for an initial relative velocity that would have resulted in a holes regime at *R*_0_ = 90.

The scaling law  was found to be confirmed by the present nanoscale droplets, which aids in bridging the gap between the micro- and the macroscale. Due to the direct accessibility of physical properties, like locally resolved temperature, the present work elucidated where and how the energy conversion takes place and gave valuable insights into the mechanisms of droplet collisions. While the validity of the scaling law on the microscale is in accordance with the macroscale, differences to the macroscopic scale were revealed since no separation but the holes regime was found for such nanoscopic droplet collisions.

## Supporting Information Available

Detailed description of the simulation setup and the analysis; process flow chart showing the setup of the droplet collision; simulation domain during the second step to simulate the equilibrated droplet; plots depicting the change in semimajor axis (top) and semiminor axis (bottom) (PDF)
Video of droplet collision with *R*_0_ = 30 and *v*_r_ = 1.0 (MP4)
Video of droplet collision with *R*_0_ = 60 and *v*_r_ = 1.0 (MP4)
Video of droplet collision with *R*_0_ = 60 and *v*_r_ = 2.0 (MP4)
Video of droplet collision with *R*_0_ = 60 and *v*_r_ = 3.0 (MP4)
