# Supplementary material for: Topologies of Nanoscale Droplets upon Head-On Collision from Large Molecular Dynamics Simulations
Source: Langmuir. 2025 Jan 8;41(2):1480–90. doi: 10.1021/acs.langmuir.4c04588 (PMC11755786; doi:10.1021/acs.langmuir.4c04588)
Supplement: Supplementary file 1 — la4c04588_si_001.pdf [file la4c04588_si_001.pdf]

SUPPLEMENTARY MATERIAL

Topologies of nanoscale droplets upon head-on collision from large molecular dynamics simulations

Leonie Tugend, Simon Homes, and Jadran Vrabec*

Thermodynamik, Technische Universität Berlin, 10587 Berlin, Germany

E-mail: vrabec@tu-berlin.de

Contents

1 Initialization and Droplet Collision Setup **2**

1.1 Step 1: Simulating the Bulk Liquid 2

1.2 Step 2: Simulating the Equilibrated Droplet 4

1.3 Step 3: Simulating the Collision Process 5

2 Sampling Tool **6**

3 Analysis Method **7**

4 Eccentricity **10**

References **10**

1 Initialization and Droplet Collision Setup

The droplet collision simulations were set up in four consecutive steps that needed to be calculated and simulated separately to obtain two droplets that can collide:

1. Simulation of an equilibrated bulk liquid
2. Cutting out and equilibrating one liquid droplet in a vapor environment
3. Duplication of the liquid droplet and its vapor environment
4. Simulation of the collision process

A code chart outlining the main functions of the droplet collision setup is given in Figure S1.

This work investigated the influence of droplet radius and initial relative velocity on the collision process. For this purpose, various initial relative velocities were simulated for each of the four droplet radii. To shorten the computing time for each collision case, the logic of the code was to first check whether a droplet of that size had already been created and saved in a checkpoint. If yes, the code skipped to immediately assigning the new relative velocities for that case and initiate *ls1 mardyn*[1] to have the droplets collide, since the first two steps are equivalent if the droplet's radius remains constant. If the droplet had not been created yet, the bulk liquid would first be equilibrated, a liquid sphere of that size cut out and then equilibrated. That new droplet would be saved in a folder for re-use in other cases, and the collision could be simulated. For each case, the Weber, Reynolds and Prandtl numbers as well as other droplet properties were calculated for evaluation.

1.1 Step 1: Simulating the Bulk Liquid

The first step in the process was the simulation of the LJTS fluid in the bulk liquid state. Initially, the molecules were placed on a face-centered cubic lattice structure resembling a solid and then equilibrated with *ls1 mardyn* until the molecules behaved as in the liquid

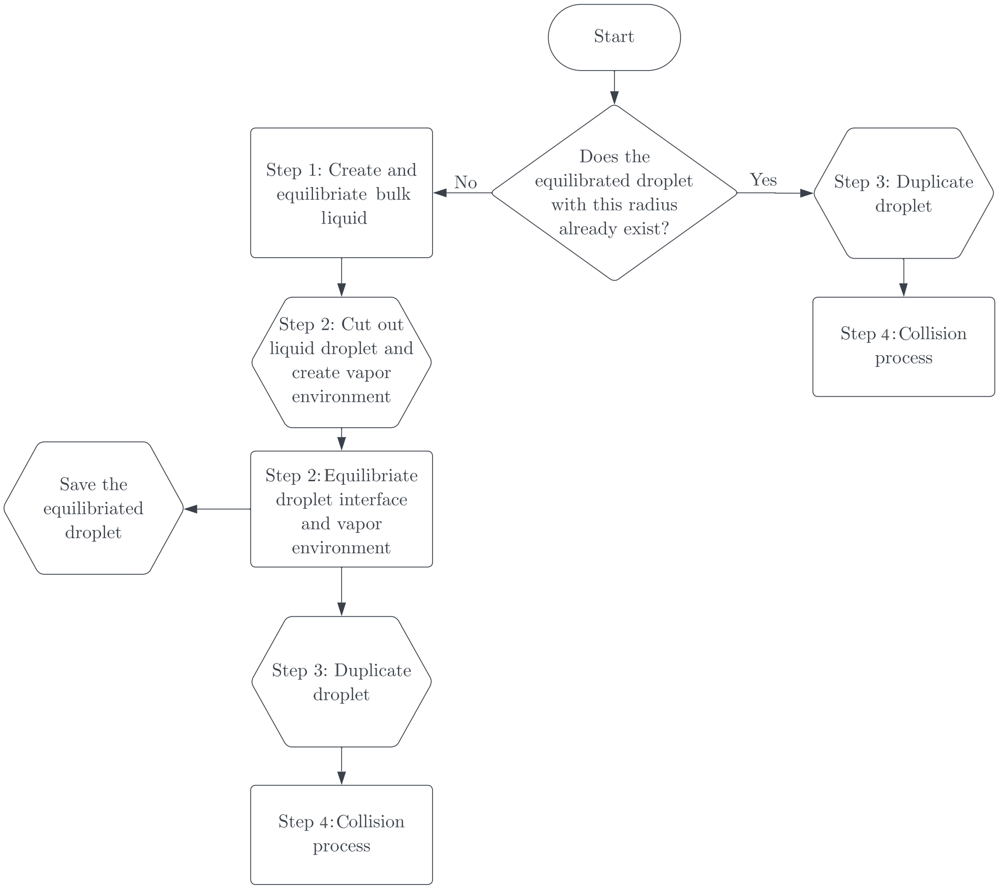

Figure S1: Process flow chart showing the function of the droplet collision *Python* code (hexagons) and the simulation runs with *ls1 mardyn*[1] (squares).

state. Figure S2 depicts the lattice structure in which the molecules were placed before equilibration and the structure that resembles a liquid after equilibration.

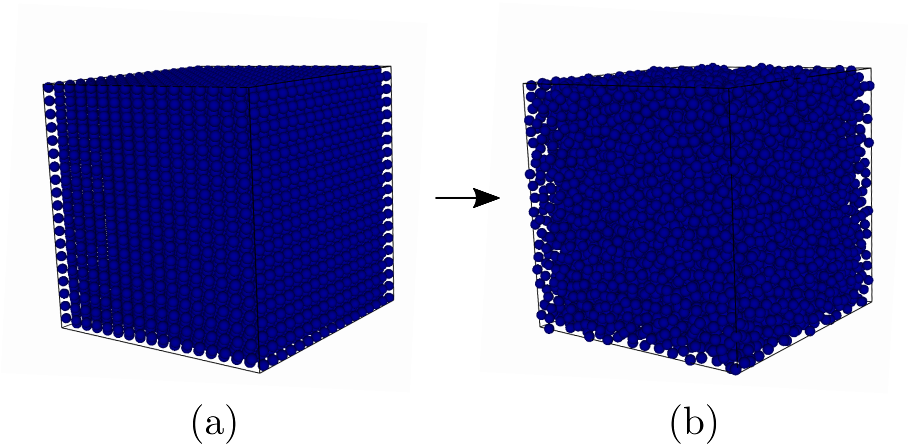

Figure S2: Simulation domain showing the grid-like structure the molecules were placed in before equilibration (a) and the liquid state after equilibration (b).

The liquid was assumed to be equilibrated when the potential energy converged to a constant, allowing this configuration to be the basis of the next simulation step in which this liquid was used to create a droplet surrounded by a coexisting vapor.

1.2 Step 2: Simulating the Equilibrated Droplet

The second step of the simulation process was to take the bulk liquid generated in step one and first cut out a sphere with the assigned radius. Since we wanted to avoid that the studied collision processes are accompanied by evaporation into vaccum, a vapor environment was also created: Most of the molecules around the liquid droplet were removed so that the droplet ambient had the saturated vapor density ρ_v and therefore resembled a gas. At this point, the *Python* code had generated a domain filled with one liquid sphere in the center surrounded by vapor. However, the liquid sphere generated by the code did not yet resemble the physical characteristics of a droplet, since the interface of the sphere was perfectly spherical. A droplet, however, has a wavy surface, which was naturally obtained by running an MD simulation in *ls1 mardyn* to equilibrate the droplet. Figure S3 shows the

interface of the droplet before and after equilibration.

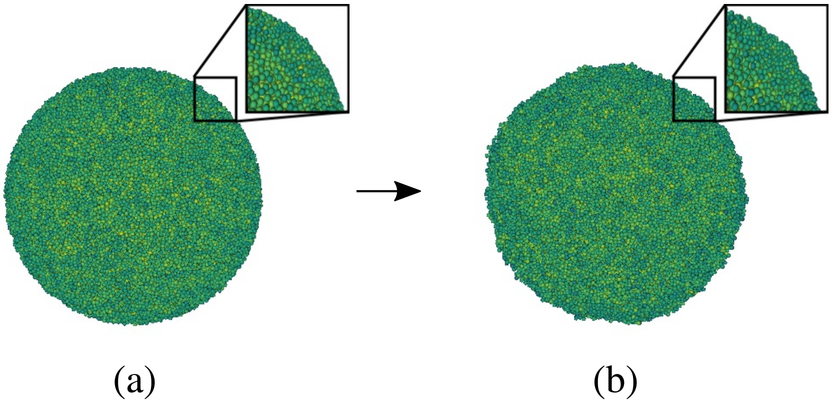

Figure S3: Simulation domain during the second step to simulate the equilibrated droplet, depicting the cut-out sphere (a) and the equilibrated droplet (b).

This can also be seen in Figure S4, which shows the difference between the interface of the sphere and the equilibrated droplet for $R_0 = 30$. Before equilibration, up to $R_0 = 30$, the density is that of the liquid droplet, which discretely falls to the vapor density for $R_0 > 30$. After equilibration, a gradual change of density from the liquid to the gas phase around $R_0 = 30$ can be observed. Due to the spherical sampling method used to measure the density throughout the domain, the statistics are not as good close to the center of the droplet, leading to fluctuating density results for $R_0 \to 0$.

After equilibration, the molecule configuration of the droplet and the surrounding vapor was written in a separate file to save simulation time for other cases with the same radius, but different initial relative velocity.

1.3 Step 3: Simulating the Collision Process

In step 3, the equilibrated droplet with the vapor environment was replicated so that two head-on droplets were placed in the center of the simulation domain and surrounded with vapor, as can be seen in Figure 1 of the main text. The size of the simulation domain was specified as $L_x = L_y = 8 \cdot R_0$ and $L_z = (10 \cdot R_0) + 10$, with 10 representing the distance between the two droplets placed in the center of the domain. This represents the initial

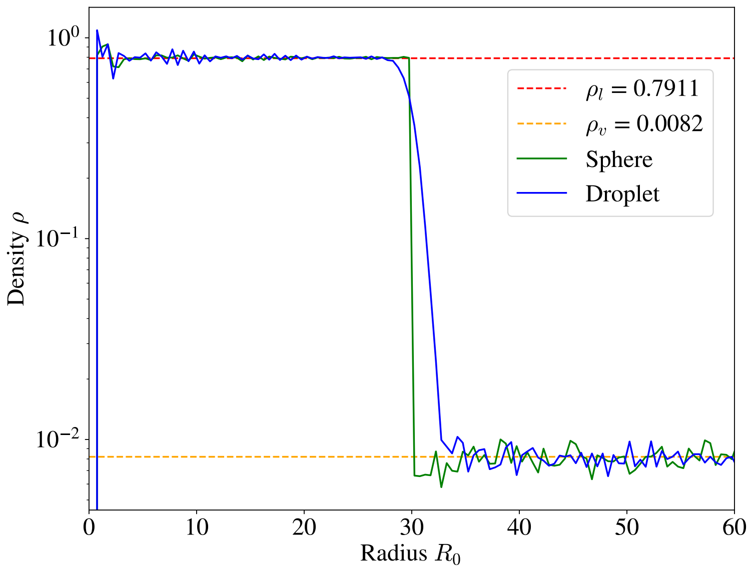

Figure S4: Density profile for $R_0 = 30$ depicting the equilibrated droplet (blue) and that of the cut-out sphere (green) in comparison to the saturated liquid density (red) and saturated vapor density (orange).

configuration of the droplets before collision. Then, *ls1 mardyn* was used to simulate the collision process. To do so, a sufficient number of time steps ($\Delta t = 0.003647347$) needed to be set so that the simulation covered a sufficiently long time interval to analyze the final state of the colliding droplets. How long the simulation needed to run, as well as the necessary domain volume, depended on the droplet size. The conditions are listed in Table SI.

Table SI: Simulation conditions for droplet radius cases.

Droplet Radius R_0	Time Steps	Domain Volume ($x \times y \times z$)	Molecule Number
30	300,000	240 $\times$ 240 $\times$ 310	3.3 $\cdot 10^5$
60	500,000	480 $\times$ 480 $\times$ 610	2.5 $\cdot 10^6$
90	600,000	720 $\times$ 720 $\times$ 910	8.3 $\cdot 10^6$
120	800,000	960 $\times$ 960 $\times$ 1210	2.0 $\cdot 10^7$

2 Sampling Tool

To further analyze the collision process, a plug-in for cylindrical sampling was used to obtain spatially resolved data on temperature, density and hydrodynamic velocity. Since this work

considered different collision cases with varying initial relative velocity, it was useful to vary the interval of time steps in which the cylindrical sampling information was written to file, so as to have a more frequent writing interval at higher velocities and fewer at lower velocities, allowing for roughly the same topology resolution regardless of velocity. This work used the initial relative velocity of $v_\mathrm{r} = 1.0$ with a write frequency of once every 500 time steps as a reference point so that the write frequency for other velocities was calculated as follows

$$\text{write frequency} = \frac{500}{v_\mathrm{r}} \,. \tag{S1}$$

3 Analysis Method

The first step to evaluate the information was to plot the density, temperature and hydrodynamic velocity profiles generated by the plug-in. Since this information was collected with a certain write frequency, it was possible to see the change of, e.g., the density of the droplets during the collision process. This was also useful as an additional method to visualize the collision, as it was possible to see the behavior of the droplets during collision through these diagrams. To identify the droplets, their interface was tracked via the density contour of the data in its accompanying binary image, since for some profiles, such as temperature or velocity, it was not as clear which data corresponded to the liquid droplet and which data related to the vapor ambient.

Once the droplet interface was identified, it was possible to calculate the axial and radial lengths of the droplets to follow the topology during collision, such as the formation of the thin ellipsoid-like structure. Figure S5 shows the lengths that were measured and evaluated in this work. The length of the droplet in the axial direction measured the distance from its center to the top edge of the contoured area, which was analogous for the radial length. Using this information, the change in axes lengths during the collision process was plotted and analyzed, as a further categorization in addition to the topology seen with *MegaMol*.[2]

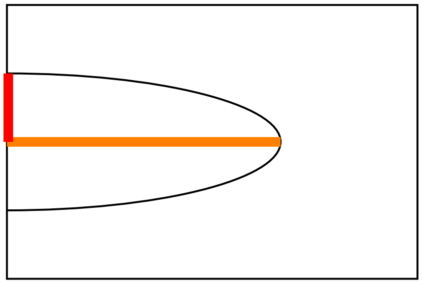

Figure S5: Schematic showing the semi-major axis a (orange) and semi-minor axis b (red) measured during a collision process.

Moreover, the eccentricity was also calculated and used as a further categorization criterion. This was done by finding the maximum point of extension of the colliding droplets in the radial direction since at this point the most pronounced ellipsoid was formed.

These calculations, along with the analysis of the topology of the droplets, allowed for classification into the collision regimes. Once this classification was done, the influences of the initial relative velocity and radius were concluded by looking at the Weber and Reynolds numbers. This then allowed for conclusions as to which conditions would lead to a given regime so that possible collision outcomes could also be anticipated.

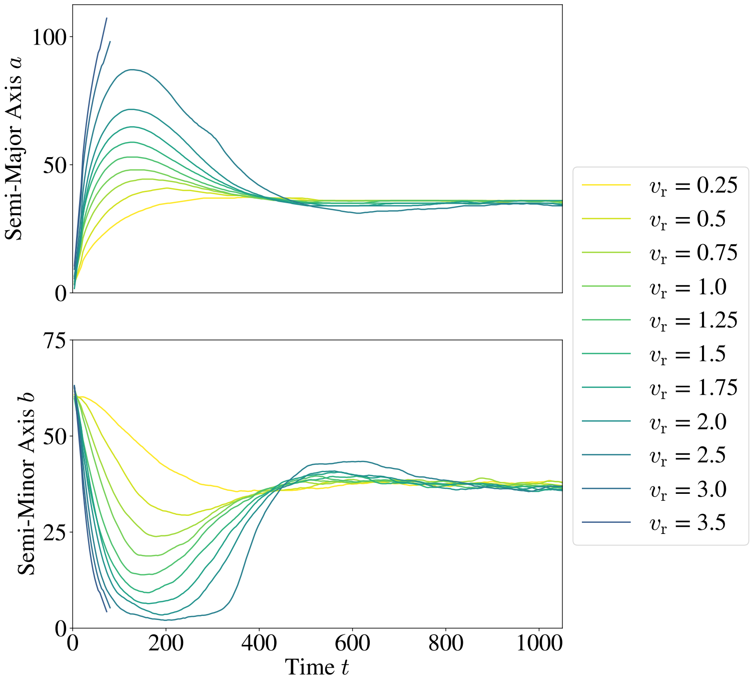

Figure S6: Plots depicting the change in semi-major axis (top) and semi-minor axis (bottom) for the $R_0 = 30$ droplets.

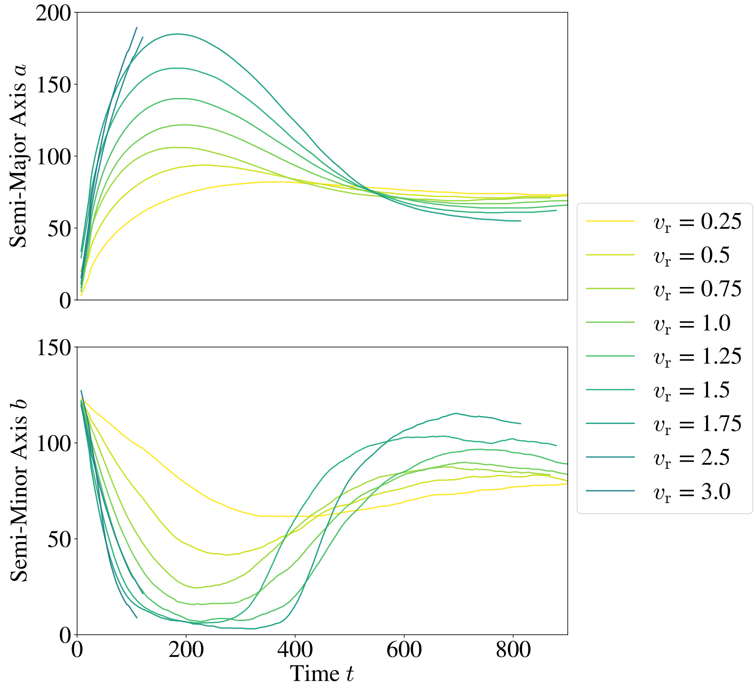

Figure S7: Plots depicting the change in semi-major axis (top) and semi-minor axis (bottom) for the $R_0 = 60$ droplets.

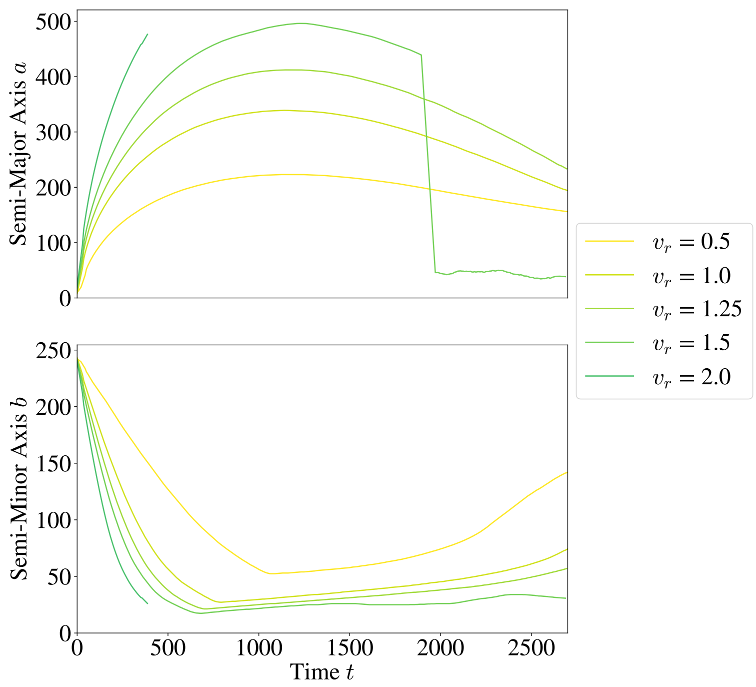

Figure S8: Plots depicting the change in semi-major axis (top) and semi-minor axis (bottom) for the $R_0 = 120$ droplets.

4 Eccentricity

Figure S9 shows the Reynolds number depending on the eccentricity. A similar picture arises compared to the Weber number as a function of the eccentricity, which is discussed in the manuscript.

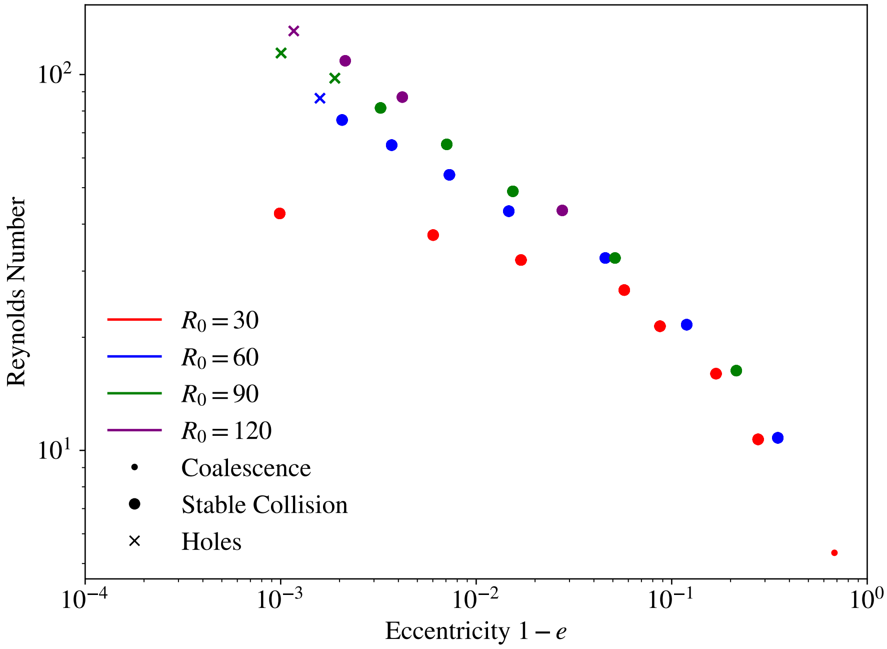

Figure S9: Reynolds number over 1 - e, where e is the eccentricity.

References

(1) Niethammer, C.; Becker, S.; Bernreuther, M.; Buchholz, M.; Eckhardt, W.; Heinecke, A.; Werth, S.; Bungartz, H. J.; Glass, C. W.; Hasse, H.; Vrabec, J.; Horsch, M. ls1 mardyn: The massively parallel molecular dynamics code for large systems. *J Chem Theory Comput* **2014**, *10*, 4455–4464.

(2) Gralka, P.; Becher, M.; Braun, M.; Frieß, F.; Müller, C.; Rau, T.; Schatz, K.; Schulz, C.; Krone, M.; Reina, G.; Ertl, T. MegaMol - A comprehensive prototyping framework for visualizations. *Eur Phys J Special Topics* **2019**, *227*, 1817–1829.
